# An efficient method for stable protein targeting in grasses (Poaceae): a case study in *Puccinellia tenuiflora*

**DOI:** 10.1186/1472-6750-14-52

**Published:** 2014-06-05

**Authors:** Yuanyuan Bu, Mengqing Zhao, Bo Sun, Xinxin Zhang, Tetsuo Takano, Shenkui Liu

**Affiliations:** 1 Key Laboratory of Saline-Alkali Vegetation Ecology Restoration in Oil Field (SAVER), Ministry of Education, Alkali Soil Natural Environmental Science Center (ASNESC), Northeast Forestry University, Hexing Road No. 26, Xiangfang District, Harbin City, Heilongjiang Province 150040, China; 2 Asian Natural Environmental Science Center (ASNESC), The University of Tokyo, Nishitokyo, Tokyo 188-0002, Japan

**Keywords:** Non-model plant, Suspension-cultured cells, Green fluorescent protein (GFP), *Agrobacterium*, Subcellular localization

## Abstract

**Background:**

An efficient transformation method is lacking for most non-model plant species to test gene function. Therefore, subcellular localization of proteins of interest from non-model plants is mainly carried out through transient transformation in homologous cells or in heterologous cells from model species such as *Arabidopsis*. Although analysis of expression patterns in model organisms like yeast and *Arabidopsis* can provide important clues about protein localization, these heterologous systems may not always faithfully reflect the native subcellular distribution in other species. On the other hand, transient expression in protoplasts from species of interest has limited ability for detailed sub-cellular localization analysis (e.g., those involving subcellular fractionation or sectioning and immunodetection), as it results in heterogeneous populations comprised of both transformed and untransformed cells.

**Results:**

We have developed a simple and reliable method for stable transformation of plant cell suspensions that are suitable for protein subcellular localization analyses in the non-model monocotyledonous plant *Puccinellia tenuiflora*. Optimization of protocols for obtaining suspension-cultured cells followed by *Agrobacterium*-mediated genetic transformation allowed us to establish stably transformed cell lines, which could be maintained indefinitely in axenic culture supplied with the proper antibiotic. As a case study, protoplasts of transgenic cell lines stably transformed with an ammonium transporter-green fluorescent protein (PutAMT1;1-GFP) fusion were successfully used for subcellular localization analyses in *P. tenuiflora*.

**Conclusions:**

We present a reliable method for the generation of stably transformed *P. tenuiflora* cell lines, which, being available in virtually unlimited amounts, can be conveniently used for any type of protein subcellular localization analysis required. Given its simplicity, the method can be used as reference for other non-model plant species lacking efficient regeneration protocols.

## Background

The subcellular localization of plant proteins is highly correlated with their functions, and as such, is a particularly relevant aspect of functional studies. In many cases, subcellular localization can be predicted *in silico* based on the primary protein sequence by exploiting the conservation of signal peptides and motifs responsible for protein targeting to different cell compartments [1]. Despite recent advances in improving the accuracy of algorithms for prediction [2,3], experimental validation of predicted localization is still the golden standard to obtain reliable functional information.

Transient gene expression assays allow for rapid and high-throughput analyses of plant genes, and thus have become widely used for characterization studies of gene function. Accordingly, several methods for transient gene expression have been developed, such as polyethylene glycol-mediated protoplast transfection [4], biolistic bombardment [5], and *Agrobacterium*-mediated transient assays [6]. In addition, stable plant transgenic lines (particularly *Arabidopsis*) expressing epitope-tagged or otherwise modified genes offer advantages in terms of ensuring a sustainable supply of plant material with homologous protein expression, the potential for mutant complementation, as well as the ability to conduct a global-scale examination throughout all tissues and cell types. Although the commonly used floral dip procedure can also be used toward this end, this procedure is more time consuming and laborious as it requires the maintenance and growth of the transgenic plants to maturity, which can take up to several weeks or longer.

**Figure 1:**
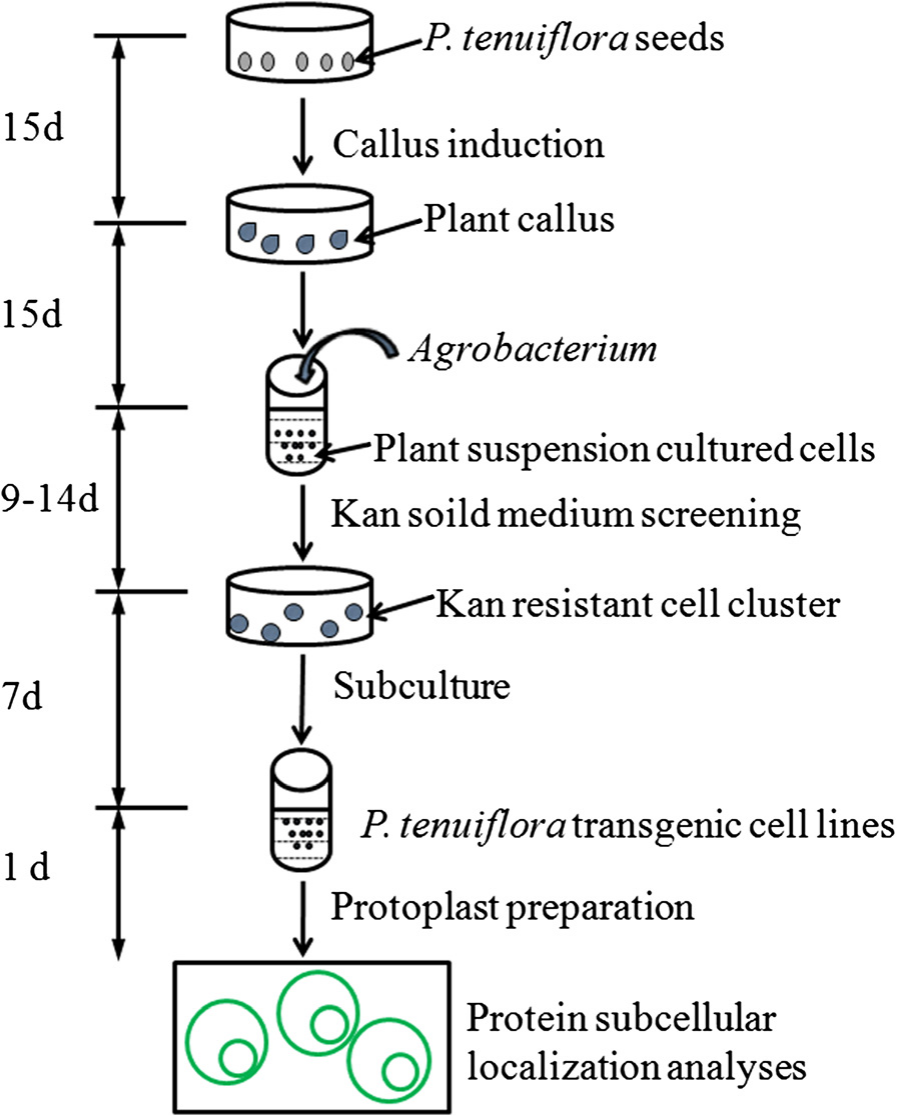
**Overview of the method for*Agrobacterium*-mediated stable transformation of*P. tenuiflora*suspension-cultured cells.** The time (in days) required for each step of the procedure is indicated to the left side of the figure.

All these methods have specific advantages and disadvantages depending on the experimental question and setup at hand. In general, however, heterologous expression systems, either transient or stable, have the major drawback of being prone to experimental artifacts. There are several challenges associated with the above-described method for determining protein subcellular localization in non-model plants, including the difficulty in obtaining transgenic plant lines and to express a protein and determine its subcellular localization in the original plant cells, because an efficient regeneration system has not yet been established for several wild plant species. To date, gene function analysis of wild plant species has been conducted using the model plant *Arabidopsis* or in yeast [7-9]; however, because these are heterologous systems, even when they successfully express certain wild plant genes, the localization of the encoded proteins might not be representative of localization in the native species [10]. Therefore, in order to best study the subcellular localization of wild plant species proteins, an effective homologous expression system needs to be established. While stable transformation of gametes or regeneration of whole plants from transformed somatic cells is routinely achieved in model plant species (e.g., by floral dipping in *Arabidopsis thaliana*), for most non-model species, the lack of suitable protocols and the phenomenon of regeneration recalcitrance make the obtainment of whole-plant transformants an arduous task. To circumvent this problem, we here propose a method for protein subcellular localization analyses that does not rely on obtaining whole-plant transformation, but instead focuses on the *Agrobacterium*-mediated stable transformation of cells in suspension-cultures (Figure 1). Plant suspension-cultured cells, a proven physiological and versatile cell system, are widely used in plant biology as a convenient tool for high-throughput functional analysis, and have also been successfully used for genetic analyses [11-16]. Here, we present a reliable and highly efficient method for stable gene expression in *Puccinellia tenuiflora* suspension-cultured cells by *Agrobacterium*-mediated transformation. We applied this method to express an endoplasmic reticulum-green fluorescent protein (ER-GFP) fusion protein in *P. tenuiflora*, a grass halophyte with extreme tolerance to alkaline soils (pH ≈ 10).

## Methods

### Materials and preparations

Wild *P. tenuiflora* seeds and *Agrobacterium tumefaciens* harboring the plasmid *pBI121-ER-GFP* were provided from the Alkali Soil Natural Environment Science Center of Northeast Forestry University, Anda practice base, Harbin, China. This plasmid contains 499 amino acids from the *PutAMT1;1* ammonium transporter from *P. tenuiflora*, which has been previously demonstrated to be localized at the nucleus periphery in correspondence with the ER and the plasma membrane [17]. *P. tenuiflora* plants were collected from an area of alkaline soil in Northeast China (Heilongjiang Province). No specific permissions were required for these activities, as sampling did not involve any endangered or protected species.

### Callus induction and subculture training

*P. tenuiflora* seeds were soaked in distilled water at 4°C for 1–2 d. After the seeds were dried and soaked in 75% alcohol for 1 min, they were rinsed 3–4 times with sterile water, then with 10% sodium hypochlorite for 30 min, and finally with sterile water 3–5 times. The calli were induced on the disinfected dry seeds after inoculation in the induction medium, which comprised Murashige and Skoog (MS) medium containing 0.5 g/L proline, 0.5 mg/L glutamic acid, 30 g/L sucrose, 2.0 mg/L 2,4-dichlorophenoxyacetic acid (2,4-D), and 0.8% agar (pH 5.8) at 25°C under illumination of 80 μmol/(m^2^ · s) for 12 h/d. Calli were obtained after 2 weeks. Weakly yellow, loose calli were selected for subculture training.

### Establishment of the system of *P. tenuiflora* suspension-cultured cell system

The selected calli were cultured at 22°C with shaking at 120 rpm in the dark. After culturing for 1, 3, 5, 7, 9, 11, 13, 15, 17, 19, or 21 d, the evenly suspended cells were harvested. Simultaneously, for subculture training, 10 mL of suspension-cultured cells (dry weight approximately 40 mg) were inoculated in liquid MS medium with different nutrient conditions: 1) different types of nitrogen nutrients, including 1/2 MS (i.e., half concentration of regular MS), MSN (i.e., half the ammonium nitrate amount of MS), MSK (i.e., half the potassium nitrate amount), MSE (i.e., additional 0.5 mg/L glutamic acid), MSP (i.e., additional 0.5 g/L proline), MSPE (i.e., 0.5 mg/L glutamic acid and 0.5 g/L proline), MSCH (i.e., 0.6 g/L casein acid hydrolysate), and MSPECH (i.e., 0.5 mg/L glutamic acid, 0.5 g/L proline, and 0.6 g/L casein acid hydrolysate); all the different media contained 2.0 mg/L 2,4-D, and 30 g/L sucrose (pH = 5.8); 2) different concentrations of sucrose, including 2.0 mg/L 2,4-D, and 0, 10, 20, 25, 30, 35, 40, or 50 g/L sucrose at pH = 5.8; 3) the same hormone at different concentrations: MSPECH + 0, 1.0, 2.0, 3.0, or 4.0 mg/L 2, 4-D and 30 g/L sucrose at pH = 5.8; 4) different pH: MSPECH + 2.0 mg/L 2, 4-D and 30 g/L sucrose at pH = 3.8, 4.8, 5.8, 6.8, 7.8, 8.8, 9.8, or 10.8. All media were tested at 22 ± 1°C with shaking at 120 rpm in the dark. After culturing for 15 d, the suspension cultures (50 mL) were centrifuged at 2000 rpm for 5 min, then dried at 70°C for 24 h and weighed; this process was repeated three times.

### Effect of acetosyringone (AS) on transformation

Three-day-old *P. tenuiflora* suspension-cultured cells were used to test the effects of AS on the transformation process. AS (100 mg/L) was added to the culture medium to create our subculture, while the cells in the culture medium without AS were used as the control.

### Isolation of protoplasts from *P. tenuiflora* suspension-cultured cells

Five-day-old *P. tenuiflora* suspension-cultured cells (5 mL) were centrifuged at 800 rpm for 5 min, and the precipitated cells were washed and centrifuged again at 800 rpm for 5 min. The newly precipitated cells were weighed and incubated in 0.05 mM MES (pH 5.8) containing 2% cellulose enzyme, 1% pectinase, 0.2 M CaCl_2_ · 2H_2_O, and 0.6 M mannitol for 3–4 h at 28°C with gentle shaking. After the enzymatic digestion, the cells were centrifuged at 100 rpm for 5 min and the supernatant was discarded. The cells were resuspended in 2 mM MES (pH 5.7) containing 154 mM NaCl, 125 mM CaCl_2_, and 5 mM KCl for 30 min. Protoplasts were harvested by centrifugation at 1000 rpm for 5 min, washed three times using the resuspension solution, and then solubilized in the solution containing 0.16 M mannitol and 0.02 M CaCl_2_ · 2H_2_O.

### Transformation of *P. tenuiflora* suspension-cultured cells

Two-week-old suspension-cultured *P. tenuiflora* cells (5 mL) were inoculated into 20 mL of freshly modified MS medium containing 100 mg/L AS. After 3 d, the cells were co-cultured with *Agrobacterium* at optical density at 600 nm (OD_600_) of 0.5, 1.0, 1.5, or 2.0 with shaking at 120 rpm and maintained at 22°C in the dark for 3 d. Then, the cells were filtered over nylon nets and washed with 200 mg/L cefotaxime (Cef) in sterile water. After being transferred to the liquid medium for 24 h, 1 mL suspension-cultured cells was inoculated into the modified MS medium containing 2.0 mg/L 2,4-D, 30 g/L sucrose (pH = 5.8), additional 0, 25, 50, 75, or 150 mg/L kanamycin (Kan), and 0, 100, 200, 300, 400, or 500 mg/L Cef at 25°C in the dark. Subsequently, the cluster of cells was transferred into 50 mL of liquid medium containing Kan and Cef and incubated at 22°C with shaking at 120 rpm in the dark for 9–14 d to obtain the transgenic suspension-cultured cells. Finally, the cells were harvested and dried at 70°C for 24 h. The dry weight was measured three times.

### GFP fluorescence observation

Green fluorescence of *P. tenuiflora* transgenic cells was observed using a stereomicroscope (Olympus, SZX9, Japan). The detection of the GFP signals was carried out using a laser-scanning confocal imaging system (Olympus Fluoview, FV500, Japan).

## Results and discussion

Most of the currently available methods for protein subcellular localization can be readily applied to the majority of model plant species such as *Arabidopsis* and tobacco, which could, in principle, lend themselves as heterologous recipients for localization studies of proteins from non-model plant species [18,19]. However, the relatively high failure rates reported in studies adopting such heterologous approaches (e.g., for *Arabidopsis* proteins expressed in tobacco) indicate that homologous expression systems are preferable whenever possible [10,20]. The lack of established regeneration protocols for the large majority of non-model plant species hinders the application of whole-plant stable transformation methods for protein subcellular localization studies, which rely mainly on transient transformation approaches. The latter methods, however, are limited with respect to the level of detail to which localization can be assessed, as subcellular fractionation or sectioning and immunodetection methods cannot be applied, thus seriously preventing analyses of functional characterization of genes in non-model species. Therefore, adaptation of methods for the stable transformation of cell cultures that are currently applied mainly for functional studies [21,22] to protein subcellullar localization analyses would provide two main advantages for cases dealing with non-model species: (1) the faithful representation of native localization patterns that are typical of homologous systems, and (2) the versatility, robustness, and virtually unlimited amount of material available for characterizing stable transformation methods. As a case study for the development of such an approach, we chose to use *P. tenuiflora*, a non-model halophite grass belonging to the Poaceae family that has been the subject of several previous studies owing to its ability to grow in soils with extremely high salinity and pH [23-26]. Given that *P. tenuiflora* is recalcitrant to regeneration, it can be conveniently used as a representative case for the proof of concept of the novel method.

### Establishment of suspension cell lines of *P. tenuiflora*

**Figure 2:**
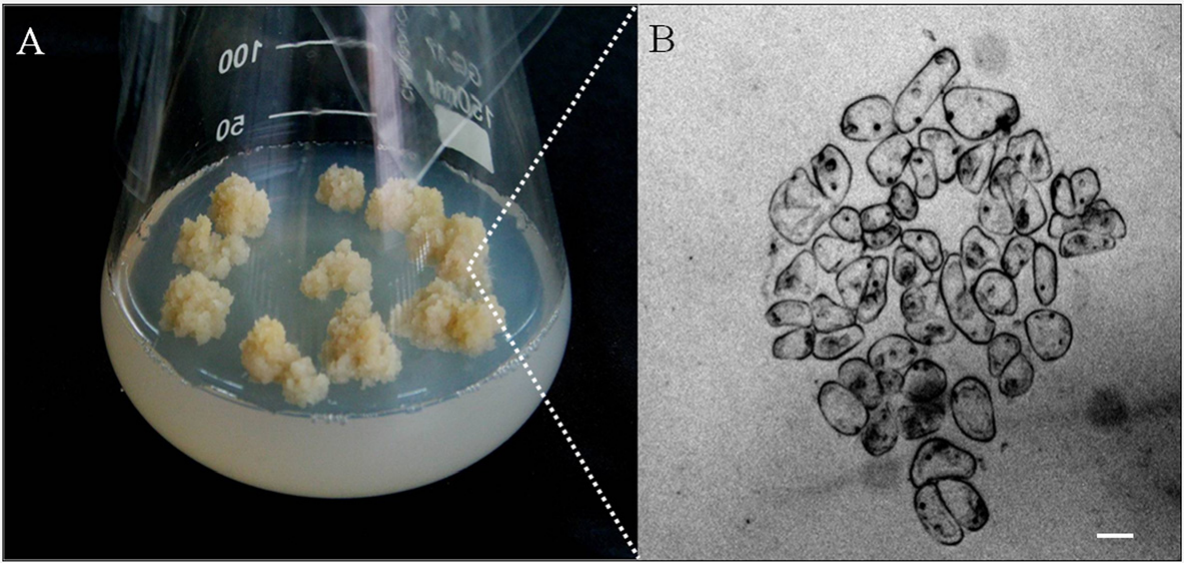
**Callus induction in*P. tenuiflora*. (A)** A friable and yellow callus of *P. tenuiflora* suspension cultures. **(B)** Magnified image of the single cells, obtained using a microscope, showing no visible intercellular aggregations and clear nuclei. Scale bar = 10 μm.

To establish *P. tenuiflora* suspension-cultured cells, a callus was induced by dedifferentiation of mature *P. tenuiflora* seeds. In brief, among the calli with different morphologies induced on the seeds of *P. tenuiflora*, we chose a yellow, loose callus (Figure 2A) with a clear nucleus and without visible intercellular aggregations (Figure 2B) for subculture. Unlike the standard method used for filtering rice [21], dispersed cells were obtained by slight grinding of the callus. The obtained cell clusters were mixed with liquid culture medium and cultured for 15 d. The culture conditions were optimized to produce the suspension-cultured cell system of *P. tenuiflora* as described below.

#### Optimization of the liquid medium

**Figure 3:**
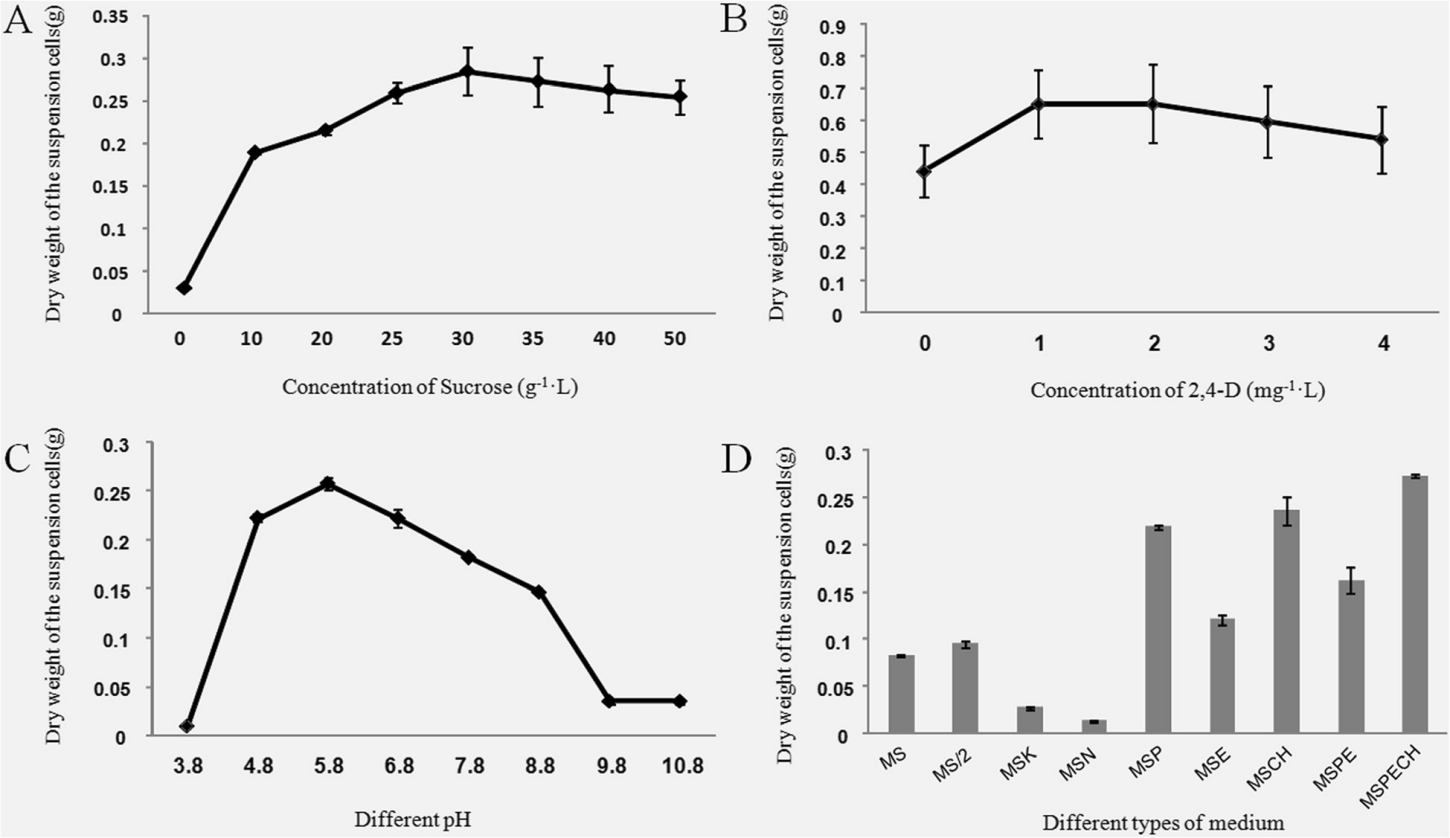
**Liquid culture medium optimization for*P. tenuiflora*suspension-cultured cells.** Dry weight of the harvested *P. tenuiflora* suspension-cultured cells at different concentrations of sucrose **(A)** and 2,4-dichlorophenoxyacetic acid (2,4-D) **(B)**, different pH **(C)**, and medium types containing different nitrogen nutrients **(D)**. The values represent the mean ± standard errors (n = 3).

We optimized the liquid medium in terms of concentration of sucrose (Figure 3A), amounts of 2,4-D (Figure 3B), pH (Figure 3C), and types of nitrogen nutrients (Figure 3D) added in a total volume of 50 mL of MS medium. After 15 d of cultivation, the dry weight of the harvested cells was measured. The dry weight of *P. tenuiflora* suspension-cultured cells was found to be highest in MSPECH medium (pH = 5.8) containing 30 g/L sucrose and 2 mg/L of 2,4-D (Figure 3), which was thus chosen as the optimum medium for the culture of *P. tenuiflora* suspension-cultured cells.

#### Growth performance of P. tenuiflora suspension-cultured cells

**Figure 4:**
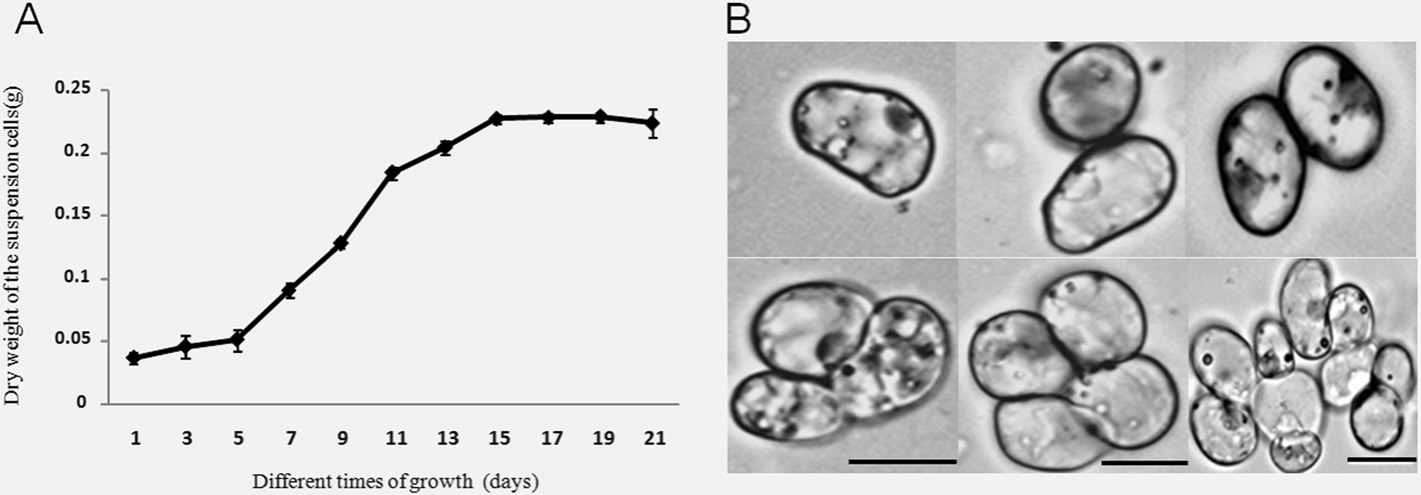
**Growth curve of*P.tenuiflora*suspension-cultured cells. (A)** Dry weight of the harvested *P. tenuiflora* suspension-cultured cells at different growth stages. **(B)** Image of *P. tenuiflora* suspension-cultured cells obtained using a microscope. Scale bar = 10 μm. The values represent the mean ± standard errors (n = 3).

In order to understand the growth dynamics of *P. tenuiflora* suspension-cultured cells, we characterized their dry weight in terms of culturing time under the optimized conditions. Figure 4A shows the growth curve of the *P. tenuiflora* suspension-cultured cells at different growth stages. Suspension-cultured cells (10 mL) were inoculated into 50 mL of MS medium, and the dry weight of the suspension was determined to be approximately 40 mg. After 5 d of cultivation, the growth rate increased and stabilized at ~15 d of cultivation, after which the cells started to show signs of aging, thus prejudicing further tests. Furthermore, as shown in Figure 4B, the cells were well dispersed, and the small cell clusters presented inclusions and uniform appearance during the 5–15 d of cultivation. Our results showed that during the 5–15 d of cultivation, the cultured cells were stable and reached a high dry weight, which were suitable conditions to perform *Agrobacterium* transformation.

#### *P. tenuiflora* suspension-cultured cells are a stable genetic transformation system

The *Agrobacterium*-mediated transformation was used to establish the genetic transformation system of the *P. tenuiflora* suspension-cultured cells. The *pBI121-ER-GFP* plasmid was used to transform the *P. tenuiflora* suspension-cultured cells. The transgenic cells were screened on a solid medium containing the appropriate marker. To ensure high transformation efficiency, the *Agrobacterium* concentration, AS effect, co-culture time during transformation, and working concentrations of Kan and Cef were optimized as described below.

#### Agrobacterium concentration

Considering that *Agrobacterium* concentration can affect the transformation efficiency, four different concentrations of *Agrobacterium* were tested: OD_600_ = 0.5, 1.0, 1.5, and 2.0. Although Kan-resistant cell clusters grew at *Agrobacterium* concentrations of an OD_600_ of 0.5, 1.0, and 1.5, the growth of the cell clusters was negatively affected at higher concentration (OD_600_ = 2.0). Therefore, we suggest that *Agrobacterium* concentration for genetic transformation should be kept as low as possible (an *Agrobacterium* concentration corresponding to OD_600_ = 0.3 was suitable for genetic transformation; data not shown). In addition, the length of co-culture during the transformation was also important for obtaining high transformation efficiency, as the bacteria were observed to be completely removed from the suspension cells after 3 d of co-culture (data not shown). Therefore, 3 d of co-culture was chosen as the optimum culturing time in order to obtain high transformation efficiency. Pandey et al. reported that optimal β-glucuronidase expression was observed in cumin embryos co-cultivated with an *Agrobacterium* cell suspension at an OD_600_ of 0.6 for 72 h [27], while the optimal transformation efficiency of suspension-cultured *Glycyrrhiza inflata* Batalin cells was achieved using an *Agrobacterium* suspension of an OD_600_ of 0.4 over 24 h of co-cultivation [22]. These results suggested that co-cultivating the lowest concentration of *Agrobacterium* possible with plant suspension-cultured cells for approximately 3 d could provide optimal conditions for achieving highly efficient transformation.

#### Optimization of the concentrations of Kan, Cef, and AS

**Figure 5:**
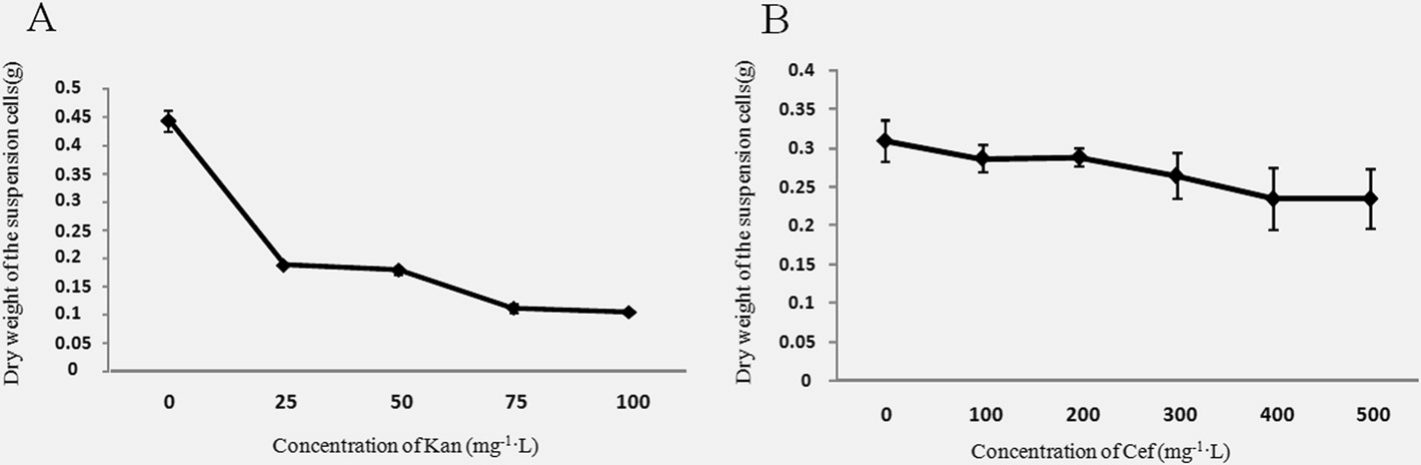
**Test of the sensitivity of*P.tenuiflora*suspension-cultured cells against different concentrations of kanamycin (Kan) and cefotaxime (Cef).** Dry weight of the harvested *P. tenuiflora* suspension-cultured cells at different concentrations of Kan **(A)** and Cef **(B).** The values represent the mean ± standard errors (n = 3).

The working concentrations of Kan and Cef were examined. Figure 5A shows that the dry weight of the suspension-cultured cells significantly decreased with increasing concentrations of Kan, and that the working Kan concentration of ~75 mg/L was most appropriate for this study. Moreover, the dry weight of the suspension-cultured cells slightly decreased with increasing concentrations of Cef. However, since the growth inhibition at 200 mg/L Cef was only slightly reduced compared to that at 300 mg/L Cef (Figure 5B), the optimum concentrations of Kan and Cef were determined to be 75 mg/L and 200 mg/L, respectively.

AS is a commonly used agent affecting *Agrobacterium*-mediated plant genetic transformation. To test the AS-mediated effects on the *Agrobacterium*-mediated genetic transformation of *P. tenuiflora* suspension-cultured cells, 3-d-old AS-pretreated suspension cells were co-cultured with *Agrobacterium* at OD_600_ = 0.5 for 3 d; non-pretreated cells were used as the control. A slight increase in the number of cell clusters from the AS-treated suspension cells on the solid medium was observed compared to that in the control (data not shown), suggesting that AS pre-treatment could improve the efficiency of infection of *P. tenuiflora* suspension-cultured cells.

### Subcellular localization of proteins

**Figure 6:**
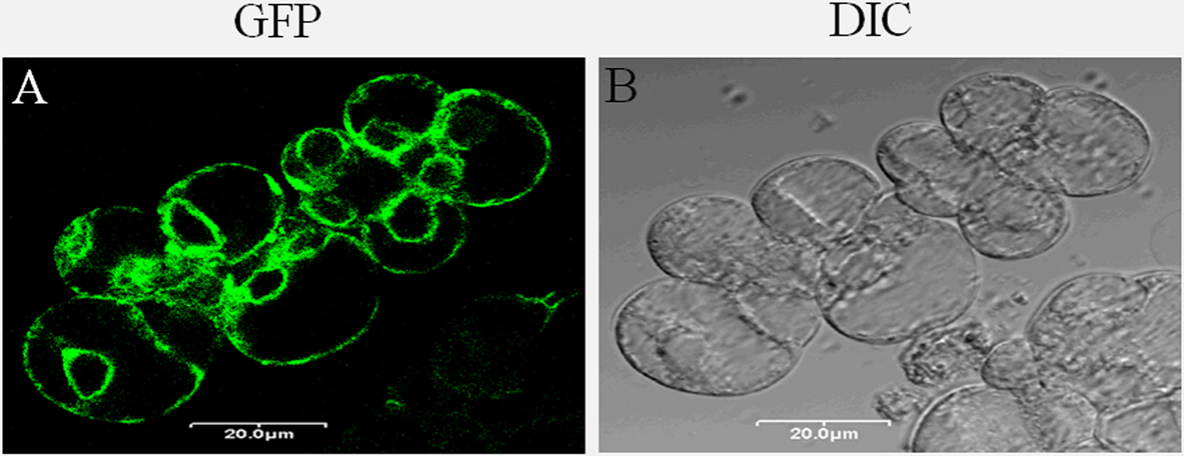
**Subcellular localization of an endoplasmic reticulum-green fluorescent protein (ER-GFP) fusion protein in*P. tenuiflora*suspension-cultured cells. (A)** GFP, **(B)** Differential interference contrast (DIC). All images were created using a laser-scanning confocal imaging system (Olympus Fluoview, FV500, Japan). GFP fluorescence was excited using an argon laser (488 nm). Scale bar = 20 μm.

In order to carry out a proof of concept study for the use of stably transformed *P. tenuiflora* suspension-cultured cells in the analysis of the subcellular localization of proteins, the plasmid *pBI121-ER-GFP*, encoding an ER-GFP fusion protein was used for genetic transformation. First, the calli were inoculated into MSPECH culture medium containing 2 mg/L 2,4-D and 30 g/L sucrose (pH 5.8) for 15 d. After 3 d, AS-pretreated cells were co-cultured with *Agrobacterium* at OD_600_ ≈ 0.3 for 3 d, and the transgenic cell lines were selected on solid MSPECH medium containing 75 mg/L Kan and 200 mg/L Cef by observing them with a stereomicroscope (Olympus, SZX9, Japan). Green fluorescent cells were manually selected for subculture to obtain highly pure *P. tenuiflora* transgenic cell lines according to previously published protoplast isolation methods, with slight modifications for improvement [28-30]. After 5 d of cultivation, the GFP signals of the transgenic protoplasts were mainly concentrated on the plasma membrane and at the ER in association with the nuclear periphery, which is consistent with the subcellular localization pattern previously established for the PutAMT1;1 protein [17] (Figure 6A-B). Given the availability of stably transformed *P. tenuiflora* cell lines expressing the PutAMT1;1-GFP fusion protein, we plan to carry out further studies using subcellular fractionation and immunogold electron microscopy to determine the localization of this protein in its native cellular environment.

Taken together, the results presented herein indicate that the *P. tenuiflora* suspension-cultured cell system could be successfully established and employed for protein subcellular localization analysis. Although previous studies reported that this approach may not be suitable for subcellular localization and other fluorescence-based analyses [31,32], we demonstrate that this optimized expression system based on *P. tenuiflora* suspension-cultured cells proved to be simple, reliable, and stable. Nevertheless, like all transformation methods relying on cells isolated from single organs without intervening regeneration of whole plants, this method can only be applied to examine the localization of proteins in cell compartments/structures that are present in the recipient cells. Therefore, the method is not suitable for studies requiring localization to the cell walls or plasmodesmata, for example, or to track protein movements in the context of organized tissues or organs. We note, however, that application of this method to other cell types deriving from different tissues such as the leaf, root, and inflorescence requires further validation to best ascertain the scope of applications of the method. In comparison to transient assays, our method is more labor-intensive and time-consuming, and thus does not lend itself readily to application in high-throughput studies [10]. However, the method does have an important advantage of providing homogeneous populations of transformed cells in virtually unlimited amounts for extended periods of time that can be used in all localization assays, which cannot otherwise be carried out on transiently transformed protoplasts owing to their complexity or particular technical requirements (e.g., cell fractionation, embedding and sectioning, immunolocalization [33,34]). Furthermore, we expect that our stable transformation method could be suitable for dynamically documenting protein re-localization through the different phases of the cell cycle or in response to environmental cues, although this was not directly tested in the present study. Most importantly, the fact that this method does not rely on whole plant regeneration makes it applicable to any non-model plant species lacking suitable regeneration protocols, which is a substantial advantage. As the method is based on simple, species-specific optimization steps of protocols that were previously developed for functional analyses in a relatively distantly related monocot species (rice), we expect that, with minor modifications, the method could be applied to several other non-model species from the Poaceae family.

## Conclusions

We have developed a rapid and stable suspension-cultured cell system for non-model plants (i.e., those lacking suitable regeneration protocols) based on an *Agrobacterium*-mediated approach. This method was successfully used for determining ER-GFP fusion protein subcellular localization in *P. tenuiflora*. Our method is simple and rapid, and is applicable to evaluating the localization of proteins only in cell compartments/structures that are present in the recipient cells.

## Abbreviations

GFP: Green fluorescent protein; ER: Endoplasmic reticulum; MS: Murashige and Skoog; 2,4-days: 2,4-dichlorophenoxyacetic acid; 1/2 MS: Half concentration of regular MS; MSN: Contains 1/2 amount of ammonium nitrate of MS; MSK: Contains 1/2 amount of potassium nitrate; MSE: Contains additional 0.5 mg/L glutamic acid; MSP: Contains additional 0.5 g/L proline; MSPE: Contains 0.5 mg/L glutamic acid and 0.5 g/L proline; MSCH: Contains 0.6 g/L casein acid hydrolysate; MSPECH: Contains 0.5 mg/L glutamic acid, 0.5 g/L proline, and 0.6 g/L casein acid hydrolysate; AS: Acetosyringone; Kan: Kanamycin; Cef: Cefotaxime; GUS: β-glucuronidase.

## Competing interests

The authors declare that they have no competing interests.

## Authors’ contributions

YB, MZ and SL designed the study. YB and MZ performed the experiments and drafted the manuscript. BS conducted the statistical analysis. XZ prepared vectors. SL and TT supervised the study and critically revised the manuscript. All authors read and approved the final manuscript.
